# Children’s Neglect of Probabilities in Decision Making with and without Feedback

**DOI:** 10.3389/fpsyg.2018.00191

**Published:** 2018-02-27

**Authors:** Anna Lang, Tilmann Betsch

**Affiliations:** Social, Organizational and Economic Psychology, Department of Psychology, University of Erfurt, Erfurt, Germany

**Keywords:** child decision making, probabilistic inference, feedback, win-stay-lose-shift, information-board

## Abstract

We investigated whether children prefer feedback over stated probabilistic information in decision making. 6-year-olds’, 9-year-olds’, and adults’ making was examined in an environment where probabilistic information about choice outcome had to be actively searched (*N* = 166) or was available without search (*N* = 183). Probabilistic information was provided before choices as predictions of cues differing in validity. The presence of outcome feedback was varied. 6-year-olds, but not 9-year-olds were over-responsive to negative outcomes leading to choices biased by recent feedback. However, children did not systematically utilize feedback in choices. Irrespective of feedback, 6-year-olds fully and 9-year-olds partly neglected stated probabilistic information in their choices. When 6-year-olds chose systematically, they only relied on invalid information, which did not maximize outcomes. 9-year-olds still applied invalid choice rules, but also choice rules based on probability. Results suggest that neglect of probabilities in complex decisions is robust, independent of feedback, and only starts to subside at elementary school age.

## Introduction

Children, like adults, must make decisions in an uncertain world. Distributions of behavioral outcomes are governed by the rules of probability. It is a developmental challenge to become sensitive and responsive to the probabilistic relations between choices and outcomes in order to achieve decision competence. Information about these relations can be conveyed prior to choices in a summarized form in terms of stated probabilities, for example by stating winning probabilities of gambles (Edwards, 1954; Kahneman and Tversky, 1979). Such information can also be gradually acquired when choices are performed and met with feedback from the environment. When sequentially sampled feedback is valid and properly stored in memory, it can provide a powerful source for subsequent intuitions and decisions (Hogarth, 2001).

There is large body of research on the utilization of stated probabilities—not only in the adult but also in the child decision making literature (e.g., Schlottmann, 2001; Levin et al., 2007a; Girotto and Gonzalez, 2008). There is also a great number of studies on experience-based decision making where children learn from feedback (e.g., Boyer, 2007; Bunch et al., 2007; Van den Bos et al., 2009). Mixed-source paradigms in which decision makers have access to both stated probabilities and feedback, however, are rare in child decision making research (Mata et al., 2011; Van Duijvenvoorde et al., 2012; Betsch et al., 2014).

In addition, so far research lacks the systematic variation of access to both kinds of information to understand their respective influence on decision making in children. As a first attempt to fill this gap, we varied the presence of feedback in an environment that provided 6-year-olds and 9-year-olds with probabilistic cues and compared their performance to adults.

### Children’s Utilization of Stated Probabilistic Information in Decision Making

In a variety of paradigms, children are confronted with stated probabilistic information in child-friendly formats, such as relative frequencies of options’ wins and losses, which they can subsequently utilize in choices. According to the classical Piagetian view, children up to 8 years of age completely lack an understanding of probability and thus the ability to properly utilize such probabilistic information (Piaget and Inhelder, 1951). However, more recent research has documented sensitivity for probabilistic information in 8-months-old infants who distinguished probable from less probable samples based on statistical properties of their environment (Xu and Garcia, 2008). Preschool-aged children are able to utilize stated probabilistic information for decisions in social contexts when choosing whom to trust or imitate (Pasquini et al., 2007; Zmyj et al., 2010); and to judge gambles with different expected values: children as young as 5 years consider winning probabilities and values and even integrate these variables in a multiplicative fashion (Schlottmann, 2001; Schlottmann and Tring, 2005).

When it is required to not only judge but to choose between two or more risky options, the findings have been strikingly different. Employing an information-board paradigm, Betsch and co-workers (Betsch and Lang, 2013; Betsch et al., 2014, 2016) assessed probabilistic inference decisions in preschoolers (6-year-olds) and elementary schoolers (9-year-olds). Preschoolers were not able to systematically use stated probabilistic information as decision weights. Moreover, only about one third of elementary schoolers was able to do so. This is consistent with studies that have shown that children up to 8 years of age do not systematically consider stated probabilistic information when choosing between lotteries (Levin and Hart, 2003; Levin et al., 2007b, but see Levin et al., 2007a). Thus, the evidence on children’s abilities to utilize stated probabilistic information for decision making is mixed. Judgment tasks so far suggest that children at preschool age consider such information (Acredolo et al., 1989; Anderson and Schlottmann, 1991; Schlottmann, 2001; Schlottmann and Tring, 2005); results in choice tasks are rather inconclusive with some demonstrating its utilization prior to school age (Levin et al., 2007a; Pasquini et al., 2007; Zmyj et al., 2010), while others do not (Levin and Hart, 2003; Levin et al., 2007b; Betsch and Lang, 2013; Betsch et al., 2014, 2016).

### Children’s Utilization of Feedback in Decision Making

Children’s ability to improve their decisions through feedback has been studied with gambling tasks in which participants repeatedly choose between options of different expected value (e.g., decks of cards), and only learn about frequencies and magnitudes of associated gains and losses through experiencing outcomes. Children up to 13 years of age typically fail to properly learn from feedback to avoid inferior options when multiple pieces of information such as gains and losses for different options have to be considered to make a choice (for a review see Cassotti et al., 2014; Defoe et al., 2014). The high complexity of this type of game presumably accounts for the poor performance (but cf. Crone et al., 2005). In simpler versions, children at preschool age learn to choose the advantageous option more frequently (Kerr and Zelazo, 2004; Boyer, 2007). When options only differ on one dimension (e.g., only gains) even 3-year-olds can adapt their choices and improve their decisions over time (Bunch et al., 2007).

Further, children’s deficits in feedback processing are, at least partly, caused by their over-responsiveness to negative outcomes. When choosing between options, children are highly sensitive toward negative feedback (Crone et al., 2005; Huizenga et al., 2007; Eppinger et al., 2009) and tend to switch behavioral responses after experiencing failure (Cassotti et al., 2011; Van Duijvenvoorde et al., 2012). In a probabilistic environment where even the best performing behavior sometimes provides negative outcomes, this tendency leads children to abandon the superior response and thus prevents optimal choice performance (see Brehmer, 1980 for similar findings in adults).

### Mixed-Information Paradigms

Studies that provide children with both stated information about probabilistic relations between choices and outcomes and self-sampled experience are rare. Mata et al. (2011) demonstrated that 9-year-olds can adapt their decision strategies to feedback in an information-board paradigm, when probabilistic information is provided before choices. Van Duijvenvoorde et al. (2012) observed children’s abilities to identify the advantageous option in a gambling task to be much improved when outcome probabilities and values were made explicit. However, even then children up to 13 years could not overcome the tendency to switch options after experiencing failure.

While these findings show that decisions can improve when the probabilistic properties of the environment are stated, research in child decision making provides little insight into how feedback influences choices that should be based on stated probabilistic information. Importantly, even in adults, feedback does not always improve decision making (see Karelaia and Hogarth, 2008 for a review) and can increase deviations from normative models (Barron and Erev, 2003; Newell and Rakow, 2007). Betsch and co-workers (Betsch and Lang, 2013; Betsch et al., 2014, 2016) found that 6-year-old preschoolers fully neglected stated probabilistic information, available as validities of different cues, and 9-year-old elementary schoolers partly did so when feedback could also be used for subsequent choices. Especially preschoolers switched between options in line with the last experienced outcome (Betsch and Lang, 2013). This leads to the question whether children’s neglect of such probabilistic information is due to the presence of feedback. As a second source of information, feedback can be used mal-adaptively by children and decrease the reliance on stated probabilistic information. If this is the case, children’s utilization of stated probabilistic information should be increased when no feedback is available. On the other hand, if feedback does not contribute to children’s probability neglect, it should be observed with and without feedback.

### Research Goal and Approach

In two studies we investigated whether feedback can account for children’s neglect of stated probabilistic information in a mixed-source paradigm. We followed the research approach from Betsch et al. (2014) in which probabilistic information was available as validities of predictive cues while feedback about decision outcomes could be experienced after each choice. Crucially, the information was redundant. Feedback reinforced the structure of the cues’ validities and reinforced both options equally. To investigate whether children’s decision making deteriorates when they can sample choices outcomes themselves, we manipulated the presence of feedback. When feedback was provided we expected children to demonstrate over-responsiveness to negative outcomes, and to prefer feedback information over cue validities, reflected in relying on a simplifying feedback-heuristic, that is, staying with one option until it fails. When feedback was absent, we expected children to increasingly utilize cue validities.

#### Research Paradigm

**FIGURE 1:**
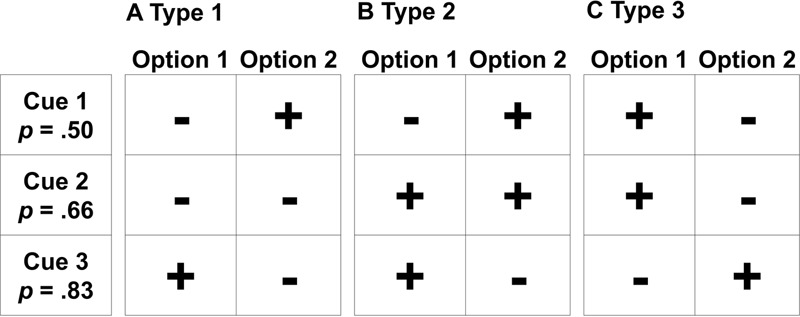
Types of prediction patterns. Each pattern was used four times in the depicted manner and four times in a mirrored version. Types were alternated starting with Type 1. In the first pattern **(A)**, the low validity cue contradicts the high validity cue’s prediction, whereas the medium validity cue is indifferent and predicts a negative outcome for both options. In the second pattern **(B)**, the medium validity cue predicts a positive outcome for both options and the low and high validity cues contradict each other. In the last pattern **(C)**, both lower validity cues contradict the high valid cue’s prediction.

In both experiments, we used a computerized version of an information-board paradigm (Mousekids) which adapted the classic Mouselab tool (Payne et al., 1988). It resembles different tasks applied in adult decision making research, such as probabilistic inferences (e.g., Gigerenzer et al., 1991), probabilistic category learning (e.g., Lagnado et al., 2006), and advice taking (e.g., Harvey and Fischer, 1997). Participants recurrently chose between two houses (i.e., decision options) in which either a treasure (i.e., positive outcome) or a spider (i.e., negative outcome) was hidden. Decision makers could inspect the predictions of three different animals (i.e., the cues), which were correct with a certain probability (i.e., cue validity, here *p* = 0.50, 0.66, 0.83). Participants were informed about cue validities in advance, and could utilize this information to maximize outcomes. Replicating the procedure from previous research (Betsch et al., 2014), participants were confronted with different patterns of cues’ predictions. Although not the focus of our analysis, this allowed us to differentiate between probability-based decision strategies (specifically WADD and LEX, see section “Choice Strategies in Children”). Each participant was confronted with each cue pattern eight times in a fixed order resulting in 24 choices (**Figure 1**). The decision environment was non-compensatory: In order to make good decisions, simple heuristics were sufficient, that is, only the predictions of the high valid cue had to be considered (Lexicographic Rule, Fishburn, 1974; Take-the-Best, Gigerenzer and Goldstein, 1996).

Following each decision, participants either received selective outcome feedback—that is, they were informed about the consequence of the chosen option—or no feedback. The probabilistic feedback structure during choices was adopted from previous research (Betsch et al., 2014) and matched each cue’s validity, that is, for example the prediction of a positive outcome by the high valid cue was correct in 83% of the trials while the prediction by the low valid cue was correct in only 50%. Either one or both options could result in positive outcomes in each trial. The feedback schedule further reinforced options equally, that is, each option provided positive and negative outcomes equally often. Thus, no option was superior. Normatively, the decisions should be based on the stated probabilistic information, that is, the cue validities and the encountered cue predictions.

#### Choice Strategies in Children

In addition to group-level analysis, the inspection of individual strategies can reveal further within-age group variability. Therefore, we analyzed individual choice behavior using an outcome-based strategy classification method to test for a variety of choice strategies in children (Bröder and Schiffer, 2003). In addition to the lexicographic rule (LEX), which predicts reliance on the high valid cue’s prediction only, we considered the weighted additive rule (WADD: integrating weighted predictions of all cues; e.g., Payne et al., 1988)^1^ and an option-based win-stay-lose-shift rule (WSLS) for the feedback conditions. According to this decision rule, the cues’ predictions are ignored and recent feedback predicts choices. A decision maker would thus stay with one option until it fails once and then switch to the other option until the next failure. This decision rule fits well with children’s tendency to switch after experiencing a negative outcome and represents a simple heuristic utilizing recent feedback. Additionally, we considered the strategy of following the low valid, but first selected cue (LVC, see section “Procedure”), since previous research has demonstrated that children sometimes rely on how much they like a cue rather than on its validity (Betsch and Lang, 2013). Finally, we tested a simple alternation strategy, consisting of systematically switching between options regardless of feedback (SW, i.e., choosing the option rejected in the last trial; Brainerd, 1981).

The considered strategies differ with regard to the amount and type of information taken into account. Two strategies are based on cue validities (LEX, WADD); one is based on recent feedback alone and can only be applied in feedback conditions (WSLS). Two rely on invalid information and neglect cue validities as well as feedback (LVC, SW). Based on prior findings (Betsch et al., 2016), we expected large differences between age groups, with the vast majority of adults, some proportion of elementary schoolers and only very few, if any, preschoolers using probability-based strategies when feedback is available. Instead we expected children to mal-adaptively use recent feedback for choices. On a group level, this would be indicated by over-responsiveness to negative outcomes, and on an individual level by the use of WSLS as a choice strategy. Without feedback we expected children of both age groups to systematically rely on cue validities in their choices. However, if probability-based strategies are equally rare, interference by feedback can be ruled out as a cause for children’s neglect of cue validities.

#### Information Presentation

**FIGURE 2:**
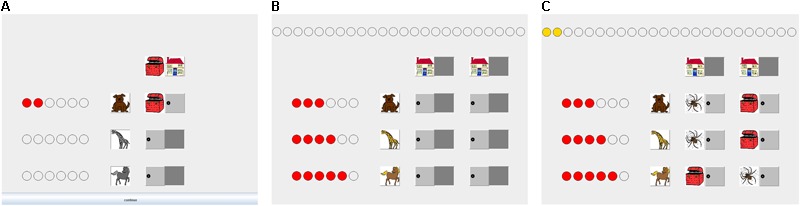
Mousekids during cue observation and choices. Figure shows a screen during the observation of cue validities **(A)**, and choices in the closed presentation format of Study 1 **(B)** and of the open presentation format of Study 2 **(C)**. Prior to choices, probabilistic information is presented as validities of different cues. Specifically, participants observe relative frequencies of correct predictions of three cues. Each animal predicts outcomes six times; for example, the first animal predicts a positive outcome (i.e., treasure). Smart points at the left of each cue serve as a visual aid of cue validity during decision making **(B,C)**. Participants then choose between two options and can inspect cue predictions. In the closed presentation format, participants uncover cue predictions before making a choice. In the open presentation format, cue predictions are uncovered. For example, participants can assess directly that the low valid cue predicts a negative outcome (spider) for the option on the left and a positive outcome (treasure) for the option on the right. At the top, a treasure point is granted for each treasure found in feedback conditions.

We conducted two studies that used the same factorial design and age groups but differed in the presentation of the cues’ predictions. In Study 1 we used a closed information-board: Predictions were hidden and active information search was required (see **Figure 2B**). Individuals could access predictions by sequentially opening doors in the board. This format is most common in comparable studies conducted with adults (e.g., Payne et al., 1988), and allowed to investigate children’s information search behavior. We tested, whether the presence of feedback affected search behavior. Specifically, we investigated whether children’s search behavior was biased by over-responsiveness to negative feedback and systematically guided by cue validities without feedback.

However, prior studies (Glöckner and Betsch, 2008; Betsch et al., 2014) have shown that the presentation format influences strategy application. Thus, in Study 2 we used an open board where all predictions are uncovered right from the start.

## Study 1: Closed Board

### Method

The study implemented a 3 (age group: preschoolers vs. elementary schoolers vs. adults) × 2 (feedback: yes vs. no) full factorial design. Pattern was varied within subjects (Type 1 vs. Type 2 vs. Type 3).

#### Sample

The sample consisted of 69 6-year-old preschoolers (28 female, *M* = 67.9 months, *SD* = 7.3), 56 9-year-old elementary schoolers (25 female, *M* = 107.4 months, *SD* = 5.7), and 56 adults (46 female, *M* = 281.18 months, *SD* = 44.66) who were randomly assigned to feedback conditions. Children were recruited from day care centers and schools with different socio-economic backgrounds from a middle-sized city in Germany. The recruitment procedure, selection of age groups, and target sample size followed considerations from prior work that used the same experimental tool (Betsch et al., 2014). Children took part in the study during a workshop at the lab and were randomly assigned to experimenters. Parents consented to participation prior to data collection. Adult participants were students of different majors.

Eight preschoolers were excluded from analyses because they did not complete the study. Five preschoolers and two elementary schoolers were excluded because they did not identify the high valid cue correctly before choices. The final sample consisted of 56 preschoolers (23 female, *M* = 68.0 months, *SD* = 7.3), 54 elementary schoolers (24 female, *M* = 107.5 months, *SD* = 5.7) and 56 adults.

#### Procedure

Each child was supervised by a trained experimenter who first introduced the child to the game’s purpose and the cues. The child selected three animals as cues. The first chosen animal was placed on the top row, the last chosen on the bottom row (see **Figure 2A**). Thus, the favored animal, that is, the first-chosen one, was always associated with the lowest validity.

##### Observing cue validities

The experimenter told the child: “Now we will find out how smart the animals are. The animals will tell you whether there is a treasure or a spider hidden in this house. But the animals are not always right. We will find out how often they are right.” She then started with the low valid cue. The experimenter opened the door next to the animal, interpreted the picture and then opened the house on top of the screen, interpreting the picture again and explained: “The dog knew that there was a treasure in the house. It was right and gets a smart point.” The experimenter clicked on the first of the smart points next to the cue, which turned red. In subsequent trials, the experimenter continued to verbalize the content of each door and house. Each time, the child was asked to indicate whether the animal should get a smart point. If the child did not answer correctly, the experimenter explained it once more. After six trials, the experimenter summarized the performance of the cue by stating that it had received three out of six smart points and continued with the next cue.

Finally, the child had observed each cue’s prediction and the particular outcome six times. This allowed us to create three cues that correctly predict outcomes with varying probability, yet perform at least at chance level (i.e., *p* ≥ 0.5). This is important, because otherwise, in a binary world, inverse probability could be used to infer outcomes (i.e., cue validities = 0.1 is equivalent to cue validity = 0.9). The first cue’s predictions were correct in three, the second cue’s predictions were correct in four, and the third cue’s predictions were correct in five out of six cases. The children translated this information into smart points, which subsequently served as a cognitive aid for representing the cue’s validity. To ensure that all children encoded differences in cue validities they were asked which animal was the smartest as a manipulation check.

##### Choice phase

The experimenter explained the game’s purpose, procedure and checked in two training trials that the child understood the choice procedure and the information-board matrix. To ensure that the children’s goal was to maximize the number of treasures found, they were informed that treasures could be traded for gifts afterward. Participants then made 24 decisions between two options represented by two houses (see **Figure 2B**). Cue predictions were covered by doors and could be uncovered before making each choice to inspect each cue’s predictions for the two options. Participants could inspect as many cue predictions as they pleased and as often as they liked. The doors covering the cues’ predictions stayed open for 3500 ms. In the feedback conditions, participants were informed about outcomes by opening the house and finding either a treasure or a spider. In conditions without feedback, the participants did not observe outcomes during the choice phase, but were informed about their performance at the end of the game. Participants were unaware of the number of decisions and prediction patterns they would encounter.

Afterward, the manipulation check was assessed a second time to rule out that children forgot about cue validities during the course of the game. Children were rewarded with two to four prizes contingent on their performance. Additionally, participants answered several questions concerning the cues and their motivation during the game, which we do not address in this paper.

##### Procedure for adults

Following previous research (Betsch et al., 2014), we used the same procedure for adults as for children with the exception that adults were informed in advance that they served as a control group for children and would receive money according to their performance (€4 on average).

### Results

#### Information Search

Children searched less information than adults did. Specifically, preschoolers on average uncovered 3.58 of the six cue predictions (*SD* = 1.77), elementary schoolers uncovered 3.72 (*SD* = 1.59) and adults 4.74 (*SD* = 1.34) predictions. In accordance with this observation, a GLM ANOVA with age group and feedback condition as between factors revealed a main effect for age, *F*(2,154) = 8.49, *p* < 0.001, $\eta_{G}^{2}$ = 0.10. Withholding feedback did not significantly affect the amount of information searched in any age group, all other *p*s ≥ 0.08.

If search behavior is guided by stated probabilistic information, the search should start on the most important information, that is, the high valid cue’s prediction. We analyzed the frequency of searches starting on the high valid cue in a GLM ANOVA with age and feedback as between factors. Adults’ searches started in 17.63 (*SD* = 9.24) of the 24 decisions on the most valid cue, while children did not systematically start their search on the high valid cue, preschoolers, *M* = 9.72, *SD* = 6.19; elementary schoolers, *M* = 11.29, *SD* = 6.21; *F*(2,154) = 18.25, *p* < 0.001, $\eta_{G}^{2}$ = 0.19. However, large variability in search behavior suggests that individuals in each age group applied different search strategies. Most importantly, searches did not start more often on the high valid cue without feedback in any age group, all other *p*s ≥ 0.16.

To test whether children’s over-responsiveness to recent negative feedback biased their information search, we compared how often it started at the non-chosen option after a negative and after a positive outcome in feedback conditions (*OR* = percentage of searches starting at the non-chosen option after losses/percentage of searches starting at the non-chosen option after gains). If children were biased by negative feedback, they should more often start their search at the non-chosen option after failure (*OR* > 1). This was only the case in preschoolers, *OR* = 1.39, 95% Bootstrap CI [1.10, 1.72]; elementary schoolers, *OR* = 0.94, CI [0.80, 1.07]; adults, *OR* = 0.90, CI [0.80, 1.00]. Comparison to preschoolers’ search behavior without feedback ensured that this was indeed due to experiencing feedback, *OR* = 0.98, CI [0.81, 1.15], *t*(53) = 2.21, *p* = 0.031, *d* = 0.28.

In line with previous research, children’s information search was not guided by cue validities (e.g., Betsch et al., 2014). In both child groups, information search was equally unsystematic with and without feedback. Interestingly, over-responsiveness to negative outcomes biased younger but not older children’s information search.

#### Choices

We first analyzed choices on the aggregate level with our focus on over-responsiveness to recent outcomes in feedback conditions and an increased use of stated probabilistic information, that is, cue validities, in conditions without feedback. Secondly, we analyzed individual choice patterns, expecting children to use the feedback-based choices rule WSLS with feedback and to use probability-based choice rules substantially more frequently without feedback.

##### Over-responsiveness to negative outcomes in choices

To test whether children tended to stay with an option and switch between options based on recent feedback, we compared the percentage of option switches after a negative outcome to option switches after a positive outcome (*OR* = percentage of switches after losses/percentage of switches after gains). Over-responsiveness is reflected in increased switching after losses in feedback conditions (*OR* > 1). In adults, switches were equally likely following positive and negative outcomes, *OR* = 0.96, 95% Bootstrap CI [0.87, 1.04]. Likewise, in elementary schoolers, switches were equally frequent, *OR* = 1.11, CI [0.92, 1.38]. In contrast, preschoolers switched significantly more often after losses than after gains, *OR* = 1.31, CI [1.11, 1.52]. To ensure that this was indeed a result of the experienced outcomes and not due to a strategy independent of feedback, preschoolers’ option switching without feedback served as a standard of comparison, *OR* = 1.10, CI [0.98, 1.25], *t*(54) = 1.61, *p* = 0.017, *d* = 0.5. Thus, only preschoolers’ choices were over-responsive to negative outcomes indicating a bias by feedback that was absent in elementary schoolers.

##### Choices following the high valid cue (HVC)

**FIGURE 3:**
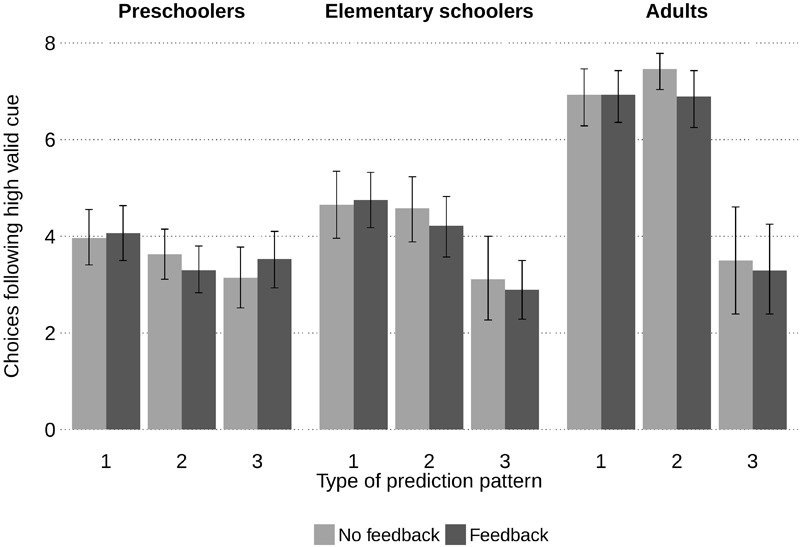
Mean frequencies of choices in line with the high valid cue’s predictions for each pattern type and feedback condition in Study 1. Chance level is four out of eight choices. Bars represent 95% bootstrap confidence intervals.

Normatively, in this environment participants should always follow the HVC. **Figure 3** depicts the mean frequencies of choices following the HVC’s prediction for each age group, separated for feedback conditions and prediction patterns. A GLM analysis of variance with the frequency of following the HVC as dependent variable, and age group, feedback condition (between) and pattern (within) as factors revealed large age and pattern effects as well as an age-pattern interaction, Age, *F*(2,160) = 57.46, *p* < 0.001, $\eta_{G}^{2}$ = 0.22; Pattern, Huynh–Feldt *F*(1.79,289.38) = 67.89, *p* < 0.001, $\eta_{G}^{2}$ = 0.20; Age × Pattern, Huynh–Feldt *F*(3.58,288.48) = 18.65, *p* < 0.001, $\eta_{G}^{2}$ = 0.12; all other *p*s ≥ 0.400.

Adults approximated normative decision making in Types 1 and 2 prediction patterns with choices following the HVC’s predictions in 7 out of 8 cases. However, when both other cues contradicted the HVC this rate dropped to about half the cases. Similarly, elementary schoolers followed the HVC’s prediction less often in Type 3 patterns and chose generally less systematically than adults but still above chance level in the first two prediction patterns. Preschoolers, in contrast, performed at chance level in each prediction pattern.

Crucially, children without feedback did not follow the HVC’s prediction more often, neither in any of the prediction patterns nor when analyzed over all 24 choices, preschoolers, *M*_No feedback_ = 10.74, *SD* = 2.68, *M*_Feedback_ = 10.96, *SD* = 3.11; elementary schoolers, *M*_No feedback_ = 12.34, *SD* = 3.88, *M*_Feedback_ = 11.86, *SD* = 3.09; adults, *M*_No feedback_ = 17.89, *SD* = 4.05, *M*_Feedback_ = 17.11, *SD* = 4.02; all *p*s ≥ 0.83. Thus, the assumption that feedback prevented children from choosing in line with normative expectations was not supported.

##### Individual choice strategies

To account for individual differences, we classified participants according to their choice behavior over all patterns using an outcome based strategy classification method (Bröder and Schiffer, 2003). Predictions were derived from five different choice models (LEX; WADD; WSLS; SW; LVC, see section “Choice Strategies in Children”). For each individual, we calculated the likelihood of the observed choice pattern under each of these models, assuming that strategies are not applied flawlessly but with a constant error maximizing the likelihood. Individuals were classified to a choice model, if their choices fitted the model predictions perfectly or if (a) the likelihood for the classified strategy was higher than for any other strategy and at least twice as high as for a Random Model (*OR* > 2), and (b) at least 66% of choices were successfully predicted by the model. Otherwise participants were classified to the Random Model. Individuals with equal likelihoods for two strategies remained unclassified. Error rates were allowed to vary over participants. **Table 1** shows the results of the classification and the mean error rates for each age group and strategy.

**Table 1:** Strategy classification in Study 1.

	No feedback		Feedback		Overall		
Preschoolers	*n* = 27		*n* = 29		*n* = 56		
	*n*	*%*	*n*	*%*	*n*	*%*	Error
LEX	-	-	-	-	-	-	
WADD	-	-	-	-	-	-	
LVC	-	-	2	6.9	2	3.6	0.29
SW	3	11.1	3	10.3	6	10.7	0.18
WSLS			1	3.4	1	1.8	0.21
Random	15	55.6	20	69.0	35	62.5	
Unclassified	9	33.3	3	10.3	12	21.4	

**Elementary schoolers**	***n* = 26**		***n* = 28**		***n* = 54**		
	***n***	***%***	***n***	***%***	***n***	***%***	**Error**

LEX	1	3.8	1	3.6	2	3.7	0.15
WADD	2	7.7	3	10.7	5	9.3	0.21
LVC	1	3.8	–	–	1	1.9	0.21
SW	4	15.4	3	10.7	7	13	0.15
WSLS			1	3.6	1	1.9	0.25
Random	15	57.5	12	42.9	27	50.0	
Unclassified	3	11.5	8	28.6	11	20.4	

**Adults**	***n* = 28**		***n* = 28**		***n* = 56**		
	***n***	***%***	***n***	***%***	***n***	***%***	**Error**

LEX	7	25.0	7	25.0	14	25.0	0.08
WADD	10	35.7	10	35.7	20	35.7	0.07
LVC	–	–	1	3.6	1	1.8	0.25
SW	–	–	1	3.6	1	1.8	0.17
WSLS			–	–	–	–	
Random	8	28.6	9	32.1	17	30.4	
Unclassified	3	10.7	–	–	3	5.4	

*Unclassified, individuals who could not be classified in one of the considered strategies; Random, random choice; probability-based choice rules; LEX, Lexicographic Rule; WADD, weighted additive rule, invalid choice rules, LVC, following low valid cue; SW, switching rule, feedback-based choice rule, only applicable in feedback conditions; WSLS, Win-Stay-Lose-Shift Rule; Error, mean error rate, that is, the mean proportion of trials in which strategy inconsistent choices were observed for the corresponding strategy and age group. For example, the error rate of 0.08 in adults being classified as LEX users means that on average strategy-incongruent choices were observed in 8%, that is, in less than two out of 24 choices. In case of n = 1, absolute values are displayed*.

The individual strategy classification largely confirmed aggregate findings. Contrary to our expectations, WSLS was not a common choice rule in either child age group, and application of probability-based strategies was not more prevalent without feedback. Most children’s choices were captured best by the Random Model (63% in preschoolers, 50% in elementary schoolers), only a minor percentage of children applied a choice rule systematically (16% in preschoolers, 30% in elementary schoolers). Intriguingly, all preschoolers that followed any strategy at all relied on invalid information with a simple switching rule being most common (11%). Elementary schoolers, on the other hand, while employing invalid strategies as often as preschoolers (17%), also and almost as often applied probability-based strategies (13%).

While the majority of adults were classified as users of probability-based strategies, 30% were classified to the Random Model. This was mainly due to inconsistent behavior by these adults in decisions with the Type 3 pattern^2^. Nonetheless, the prevalence of probability-based decision strategies was strongly determined by age, χ^2^(2, *N* = 166) = 61.42, *p* < 0.001, Cramer’s *V* = 0.61. Probability-based strategies were mainly used by adults (61%), rarely by elementary schoolers (13%), and not found in preschoolers. Complementary, invalid choice rules were not found in adults and were observed equally often in preschoolers (16%) and elementary schoolers (17%), χ^2^(1, *N* = 110) = 0.007, *p* = 0.93.

### Discussion

In prior studies, no preschoolers and only a small proportion of elementary schoolers utilized stated probabilistic information presented as cue validities in their search and choice behavior (Betsch et al., 2014). We suspected that choice feedback led children to rely on recent outcomes rather than cue validities and could thus be the reason for the observed probability neglect. If this assumption were true, over-responsiveness to negative outcomes in search and choice behavior and systematic use of feedback in terms of WSLS as a choice rule should be observed. Withholding feedback should lead to an increased utilization of cue validities in children’s searches and choices. Study 1 provided some evidence of the former but no indication of the latter. Preschoolers showed over-responsiveness in search and choice behavior; but children’s behavior was not increasingly guided by cue validities without feedback. We address both results in the General Discussion. However, we first wanted to corroborate the findings and rule out that expected differences in utilization of cue validities were disguised by the presentation format of the information-board.

Specifically, the application of choice strategies can be hampered in a closed information-board. The predictions of the cues need to be looked up sequentially and kept in memory until a decision is made. Such an environment binds cognitive resources and, hence, may impede successful strategy implementation (Glöckner and Betsch, 2008). This is especially the case in children who have demonstrated significant deficits in searching for relevant information in information-board paradigms until late elementary school age (Davidson, 1996; Katz et al., 2010; Betsch et al., 2016). For example, in order to apply a normatively correct strategy like LEX, children have to look for the prediction of the HVC first and keep it in mind until they choose an option. Even when applying WSLS, where a search of cue predictions is not necessary, the delay between choices could nevertheless hinder successful strategy application. Accordingly, the lack of usage of WSLS in feedback conditions and of probability-based strategies without feedback might have been a consequence of a decision environment that impeded application of strategies.

## Study 2: Open Board

We replicated the first study in a decision environment without constrained access to information to facilitate the application of choice strategies and thereby the detection of differences in strategy application between feedback conditions.

### Method

The design, stimulus material, and procedure were identical to Study 1, with the exception that the information-board matrix in the choice phase was open so that all predictions could be inspected at once for every decision (see **Figure 2C**). Search data are therefore not available.

#### Sample

The sample consisted of 80 preschoolers (49 female, *M* = 69.2 months, *SD* = 5.3), 62 elementary schoolers (28 female, *M* = 104.4 months, *SD* = 4.7), and 53 adults (39 female, *M* = 258.59 months, *SD* = 31.96). Two preschoolers, one elementary schooler, and one adult had to be excluded because they did not complete the study. Seven preschoolers and one adult did not pass the manipulation check. The final sample consisted of 71 preschoolers (46 female, *M* = 69.1 months, *SD* = 5.3), 61 elementary schoolers (27 female, *M* = 104.4 months, *SD* = 4.7), and 51 adults (37 female, *M* = 267.0 months, *SD* = 31.2). The recruitment procedure was the same as in Study 1.

### Results

#### Over-Responsiveness to Negative Outcomes in Choices

Again, only preschoolers switched options more frequently after negative than after positive outcomes, *OR* = 1.29, 95% Bootstrap CI [1.11, 1.49]; without feedback, *OR* = 1.07, CI [0.99, 1.16]; *t*(69) = 2.02, *p* = 0.003, *d* = 0.49. Elementary schoolers in feedback conditions showed no such tendency, *OR* = 1.08, CI [0.95, 1.24], neither did adults, *OR* = 0.78, CI [0.68, 0.90].

#### Choices Following the High Valid Cue (HVC)

**FIGURE 4:**
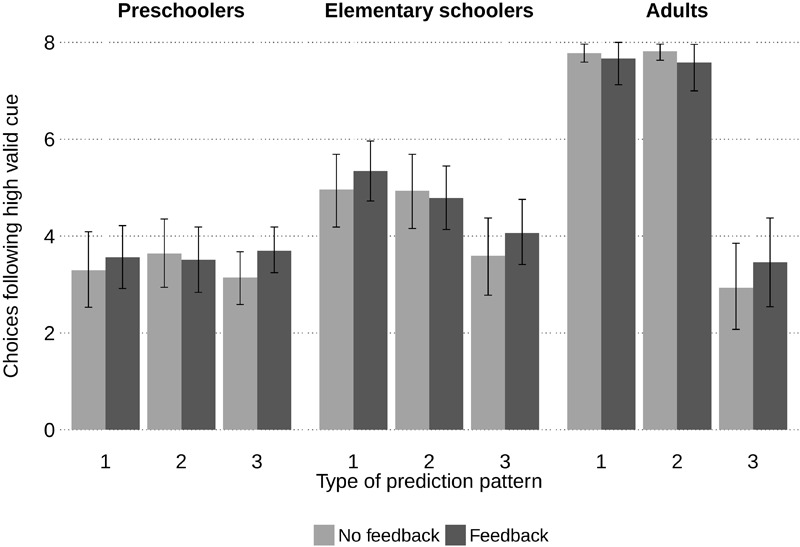
Mean frequencies of choices in line with predictions of the high valid cue for each pattern type and feedback condition in Study 2. Chance level is four out of eight choices. Error bars represent 95% bootstrap confidence intervals.

**Figure 4** depicts the mean frequencies of following the HVC’s predictions for every age group, feedback condition and prediction pattern. The results largely matched those in Study 1. Again, feedback did not affect choices in any age: not in any of the patterns nor when analyzed over all 24 choices, preschoolers, *M*_No feedback_ = 10.09, *SD* = 4.63, *M*_Feedback_ = 10.78, *SD* = 4.42; elementary schoolers, *M*_No feedback_ = 13.50, *SD* = 5.02, *M*_Feedback_ = 14.21, *SD* = 4.35; and adults, *M*_No feedback_ = 18.52, *SD* = 2.58, *M*_Feedback_ = 18.70, *SD* = 3.65; all *p*s ≥ 0.40. A GLM ANOVA resulted in similarly large age and pattern effects, and a pattern-age interaction as in Study 1, Age, *F*(2,177) = 54.65 *p* < 0.001, $\eta_{G}^{2}$ = 0.26; Pattern, Huyn–Feldt *F*(1.59,281.64) = 93,24, *p* < 0.001, $\eta_{G}^{2}$ = 0.19; Age × Pattern, Huyn–Feldt *F*(3.81,281.64) = 42.79, *p* < 0.001, $\eta_{G}^{2}$ = 0.18, all other *p*s ≥ 0.170. Adults followed the HVC’s prediction except when both other cues contradicted its prediction. The same pattern effect was observed in elementary schoolers, who followed the HVC’s prediction less systematically but above chance level in the first two prediction patterns. Preschoolers performed at chance level, regardless of the prediction pattern.

#### Individual Choice Strategies

The results of the individual strategy classification are shown in **Table 2**. Similar to Study 1, feedback did not influence the prevalence of probability-based strategies in either child age group and WSLS was used rarely.

**Table 2:** Results of strategy classification in Study 2.

	No feedback		Feedback		Overall		
Preschoolers	*n* = 34		*n* = 37		*n* = 71		
	*n*	*%*	*n*	*%*	*n*	*%*	Error
LEX	–	–	1	2.7	1	1.4	0.17
WADD	–	–	–	–	–	–	
LVC	3	8.8	4	10.8	7	9.9	0.14
SW	16	47.1	11	29.7	27	38.0	0.12
WSLS			–	–	–	–	
Random	13	38.2	17	45.9	30	42.3	
Unclassified	2	5.9	4	10.8	6	8.5	

**Elementary schoolers**	***n* = 32**		***n* = 29**		***n* = 61**		
	***n***	***%***	***n***	***%***	***n***	***%***	**Error**

LEX	4	12.5	4	13.6	8	13.1	0.12
WADD	2	6.3	–	–	2	3.3	0.08
LVC	2	6.3	–	–	2	3.3	0.17
SW	10	31.3	5	17.2	15	24.6	0.19
WSLS			1	3.4	1	1.6	0.29
Random	9	28.1	12	41.4	21	34.4	
Unclassified	5	16.5	7	24.1	12	19.7	

**Adults**	***n* = 27**		***n* = 24**		***n* = 51**		
	***n***	***%***	***n***	***%***	***n***	***%***	**Error**

LEX	3	11.1	5	20.8	8	15.7	0.04
WADD	8	29.6	9	37.5	17	33.3	0.05
LVC	–	–	1	4.2	1	2.0	0.25
SW	–	–	–	–	–	–	
WSLS			–	–	–	–	
Random	13	48.1	9	37.5	22	43.1	
Unclassified	3	11.1	–	–	3	5.9	

*Unclassified, individuals who could not be classified in one of the considered strategies; Random, random choice, probability-based choice rules; LEX, lexicographic rule; WADD, weighted additive rule; invalid choice rules; LVC, following low valid cue; SW, switching rule, feedback-based choice rule, only applicable in feedback conditions; WSLS, Win-Stay-Lose-Shift Rule; Error, mean error rate for the corresponding strategy and age group. In case of n = 1, absolute values are displayed.*

Adults applied probability-based choice strategies to a large extend (49%, LEX, WADD). However, like in Study 1, a relatively large proportion was classified to the Random Model. Again, that was mainly due to their indifferent choice behavior in Type 3 prediction patterns (43%)^3^. Among children that chose systematically, elementary schoolers used valid (16%) as well as invalid strategies (28%, LVC, SW) while preschool-aged children relied predominantly on invalid ones (48%). The prevalence of probability-based strategies was again strongly dependent on age, with 49% of adults (*n* = 25), and 16% of elementary schoolers (*n* = 10) using such strategies, but only 3% of preschoolers (*n* = 1), χ^2^(2, *N* = 183) = 43.20, *p* < 0.001, Cramer’s *V* = 0.47. Likewise, the use of invalid strategies was most common in preschoolers, (48%), still found at elementary school age (28%) and only sporadically present in adults (2%). However, in contrast to Study 1, invalid choice rules were more common in preschoolers than in elementary schoolers, χ^2^(2, *N* = 132) = 4.64, *p* = 0.031, Cramer’s *V* = 0.19.

### Discussion

In an open board, no active search processes are needed to access information. We tested whether this would foster children’s systematic utilization of the provided sources of information—either stated probabilistic information, that is, cue validities, or feedback. However, the findings of Study 2 were strikingly similar to those of Study 1. Thus, we are confident that the lack of systematic utilization of cue validities or feedback is not the result of the presentation format requiring too much of children’s limited cognitive resources. However, compared to Study 1 more children of each age group applied systematic choice rules, indicating that open presentation facilitated strategy application, but not utilization of probabilistic information or feedback.

## General Discussion

Achieving decision competence requires, as a prerequisite, that individuals develop sensitivity and responsiveness to the probabilistic relations between choices and outcomes. We employed a research paradigm in which information about these relations was provided prior to choices—predictions of cues with different validities—in addition to self-sampled feedback about the choices’ consequences. Previous studies using this paradigm found that preschoolers around the age of 6 years are virtually insensitive to stated probabilistic information in choices while a minority of 9-year-old elementary schoolers is able to utilize it in a systematic fashion (Betsch and Lang, 2013; Betsch et al., 2014, 2016). This evidence as well as the present studies contradict findings from other domains of developmental research where the utilization of stated probabilistic information prior to school age has been observed (e.g., Schlottmann, 2001; Pasquini et al., 2007). To investigate the potentially interfering effect of experience on utilization of stated probabilistic information, we manipulated the presence of feedback in a closed and open information-board paradigm. We hypothesized that experiencing decision outcomes provides an interfering second source of information and can account for children’s neglect of stated probabilistic information.

The results, however, show that the removal of feedback from the decision task did not improve children’s performance. Normative utilization of probability in choices prescribes to rely only on the high valid cue’s predictions. In either study, though, the frequency of following the high valid cue did not differ as a function of the presence of feedback. Compared to conditions with feedback, choices did not follow the high valid cue’s prediction more often without feedback in any age group; preschoolers, *d*_Study1_ = -0.08, 95% CI [-0.61, 0.44], *d*_Study2_ = -0.15, CI [-0.62, 0.31], *d*_overall_ = -0.12, CI [-0.47, 0.23]; elementary schoolers, *d*_Study1_ = 0.14, CI [-0.40, 0.67], *d*_Study2_ = -0.15, CI [-0.65, 0.36], *d*_overall_ = -0.01, CI [-0.38, 0.35]; adults, *d*_Study1_ = 0.19, CI [-0.33, 0.72], *d*_Study2_ = -0.06, CI [-0.61, 0.49], *d*_overall_ = 0.07, CI [-0.31, 0.45]. Individual analysis of choice patterns further showed that the prevalence of probability-based strategies was not increased without feedback and that when feedback was available children did not use a feedback-based choice strategy. Tracing information search behavior in Study 1 demonstrated additionally that younger children’s over-responsiveness to negative outcomes also affected their search behavior, although searches were equally unsystematic without interfering feedback.

We conclude that children did not neglect stated probabilistic information because they preferred feedback as a source of information. Consequently, the reasons for children’s probability neglect must lay elsewhere (see section “Robustness of Probability Neglect”). Our two studies yielded three important new insights:

### Over-Responsiveness to Negative Outcomes But No Systematic Use of Feedback-Based Strategies

Children overreact to negative feedback and tend to switch responses. This behavior has been attributed to deficits in inhibitory control (Crone and van der Molen, 2007; Van Duijvenvoorde et al., 2012); and linked to the assumption, that negative consequences lead to an affective reaction to avoid this option, which must be actively overruled (Damasio, 1994, but see Brehmer, 1980). At the age of nine, children in our studies no longer showed over-responsiveness. This contrasts prior findings of over-responsiveness to negative outcomes until late school age (Cassotti et al., 2011), even when winning probabilities of options are stated explicitly (Van Duijvenvoorde et al., 2012).

Importantly, although over-responsiveness biased preschool-aged children’s search and choice behavior, they did not strategically utilize recent outcomes and consistently rely on an option-based Win-Stay-Lose-Shift rule for choices, which solely considers recent feedback. On individual level Win-Stay-Lose-Shift did not fit children’s choice patterns when compared to other plausible models of choice. This apparent contradiction underlines the important distinction between data analysis at the aggregate and the individual level. It is still possible, that children applied a more sophisticated form of Win-Stay-Lose-Shift as a feedback-based strategy, for example switch and stay in relation to the option’s expected value calculated over several trials (e.g., Worthy and Maddox, 2014). However, application of such a strategy is inconsistent with the overall lack of differences between feedback conditions on both aggregated and individual level.

### Robustness of Probability Neglect

In line with previous findings, we observed large differences between age groups in the utilization of stated probabilistic information for choices in both studies. The majority of adults used strategies based on stated probabilistic information such as the Lexicographic or Weighted Additive Rule consistently while only few elementary schoolers and only one preschooler did so. Previous studies with the same paradigm have found that such probability neglect is only marginally affected by variations of the decision environment such as information search constraint (Betsch et al., 2016) or lure information (Betsch and Lang, 2013). The present findings further underline the robustness of probability neglect in children’s risky decision making. Across two decision environments—one requiring an active search for cue predictions, the other displaying all cue predictions without search—preschool-aged children at the age of six were unable to utilize stated probabilistic information to adapt choices, whereas 9-year-old elementary schoolers were partly able to do so. Further, withholding feedback had no facilitating effect on the utilization of stated probabilistic information at the aggregate or the individual level. Thus, probability neglect is not a consequence of the mixed-source paradigm which offers feedback as a second and possibly preferred source of information.

Our findings contribute to the complex picture of children’s ability to utilize stated probabilistic information for decisions and are in line with findings from gambling studies that highlight children’s deficits until late school age (Levin and Hart, 2003; Levin et al., 2007b). However, there is also contradicting evidence stemming from selective trust tasks (Pasquini et al., 2007), judgments tasks (Schlottmann, 2001), and low-complex experienced-based gambling tasks (Bunch et al., 2007), in which utilization at preschool age was observed. Although these paradigms differ much in terms of information presentation and complexity, they all share that probabilities are directly assigned to options. The challenge for children is to make advantageous choices between these options. In a probabilistic inference task, however, the relations between options and outcomes are moderated by cues, to which these probabilities are assigned. Therefore, they pose a greater challenge to children’s conceptual understanding of probabilities, which might overburden them and explain the contradictory results. Probability neglect in children might therefore strongly depend on specifics of the probabilistic environment created by the research paradigm.

### Development of Decision Strategies

Our results corroborate prior findings regarding the prevalence of probability-based strategies in individuals of different age groups (e.g., Mata et al., 2011; Betsch et al., 2014). At the age of six, children do not rely on probability-based choice rules, at the age of nine this ability is still emerging. This is in line with research from other areas that suggests a strong developmental improvement in cognitive strategy use during that period (see Björklund and Causey, 2018 for an overview). Yet, while most children neglected both sources of potentially valid information—stated probabilistic information and feedback—many nonetheless did not chose randomly between options. Instead, they systematically relied on invalid information, that is, information that was not useful to maximize choice outcomes, such as, which cue was liked the most and which option had been chosen in the preceding decision. This is not only interesting from a theoretical point of view, but holds important implications for the improvement of decision quality in children. Rather than just introducing or teaching appropriate strategies, children’s current strategies must be directly addressed and revealed as inappropriate.

### Implications of Individual Strategy Classification

In each age group we observed rather large proportions of individuals that could not be classified to a decision strategy (i.e., Random). This finding has two important implications. First, it shows that adults’ decision behavior is a more appropriate benchmark for children’s decisions than normative standards, that is, ideal choices under all circumstances. Second, though, it points to a limitation of our strategy classification method. Many adults remained unclassified because they choose indifferently in Type 3 prediction patterns. We cannot rule out, however, that these participants used different strategies for different prediction patterns, which the applied method cannot detect, or might have used decision strategies not considered a priori. For example, participants might have applied an Equal-Weight Rule (e.g., Payne et al., 1988), which prescribes to follow the majority of predictions and, in case of a tie, to guess between options. This would lead to guessing in two thirds of the investigated choices and choices in line with the majority in Type 3 prediction patterns. We cannot rule out that single participants might have used this strategy; however, choices in Type 3 prediction patterns are not in line with this model.^4^ It is therefore unlikely, that it was widely used in any age group.

Further, it is conceivable, that participants classified to WADD instead used a compound-strategy that favors the option with more positive predictions and only in case of indifferences considers cue validities. Although, this strategy would lead to exactly the same choices as WADD, the underlying cognitive steps are quite different. Nonetheless, it would still rely on the probabilistic cues. As our main focus was to differentiate choice rules based on which kind of information was used—probabilities, feedback, or invalid information—the possibility of a compound-strategy does not alter our interpretation of the results.

### Beneficial Aspects of Feedback

This paper aimed to investigate potentially interfering effects of feedback on children’s utilization of probabilities. However, feedback does not have to be harmful. A study by Mata et al. (2011) suggests that 9-year-olds can adapt decision strategies via feedback learning and our own results demonstrate that even 6-year-olds are generally responsive to feedback in probabilistic inferences, even if this resulted in over-responsiveness to negative outcomes. Unfortunately, in order to reveal such beneficial aspects of feedback, the number of decisions has to be large enough to enable learning in all age groups while learning rates may vary with age. Since the number of decision was limited in our studies, no conclusion about potential beneficial aspects of feedback can be drawn.

## Conclusion

So far, research had demonstrated that children up to late school age fail at utilizing probabilities in a complex environment with multiple predictive cues (Betsch and Lang, 2013; Betsch et al., 2014, 2016). Our studies replicated these findings and ruled out the interference of feedback information as a cause for this probability neglect. Below school age, children’s choices are not ruled by probabilities. If at all, irrelevant features of the task guide their choices. At elementary school age, utilization of probabilities is only emerging, yet not consolidated in all children. At that age, children are still far from being competent when decisions are risky and complex.

## Ethics Statement

The studies were carried out in accordance with the recommendations of Ethical Standards in Research of the Society for Research in Child Development. Parents gave informed consent for child participants, children could choose to discontinue participation at any time. Adult subjects gave written informed consent in accordance with the Declaration of Helsinki. The protocol was approved by the Research Ethics Committee of the University of Erfurt.

## Author Contributions

AL and TB contributed to the study design and approved the final version of the manuscript. AL performed the data collection, analysis, interpretation, and drafted the manuscript. TB provided the critical revisions.

## Conflict of Interest Statement

The authors declare that the research was conducted in the absence of any commercial or financial relationships that could be construed as a potential conflict of interest.

## Footnotes

1 For WADD all cue predictions should be weighted by cue validities (CV) and added up to determine the expected value for each option. In order to achieve a non-compensatory decision environment, we included a cue whose predictions were at chance level and could therefore be ignored. However, whether CVs are used as weights or must be transformed is discussed controversially [e.g., correcting for chance level, CV-0.5, Jekel et al., 2012; Hilbig and Moshagen, 2014; or log-transforming, log(CV/1-CV), Lee, 2014]. For determining predictions of WADD we used CVs without any correction as weights, thus WADD would predict following the HVC in the Types 1 and 2 predictions patterns but not in Type 3 where both other cues overrule predictions of the HVC (Rieskamp and Hoffrage, 1999). Based on previous research (Betsch et al., 2014) we assumed that a substantial proportion of adults would apply an uncorrected form of WADD in this decision environment although it contradicts normative expectations, see also Footnote 2 and 3.

2 The vast majority of adults classified to the Random Model did not choose randomly in all types of predictions patterns. Only in Type 3 patterns their choices followed the HVC at chance level, *Mdn* = 4, interquartile range (IQR): 2.5–6; while in Type 1 and 2 they were closely aligned with normative choice behavior, *Mdn*_Type1_ = 7, IQR: 5–8; and *Mdn*_Type2_ = 8, IQR: 6–8 (see also Footnote 1). However, since the classification method assumes a constant application error in all prediction patterns, and therefore cannot account for deviations in only one pattern, these adults were not classified to any of the choices models.

3 For adults classified to the Random Model, *Mdn*_Type1_ = 8, IQR: 8–8; *Mdn*_Type2_ = 8, IQR: 8–8; and *Mdn*_Type3_ = 3, IQR: 2–5.

4 In two thirds of all choices, that is, prediction patterns Types 1 and 2, Equal Weight and Random predict guessing, and can therefore not be reliably distinguished from each other on an individual level. In Type 3 prediction patterns, Equal Weight prescribes following the majority (e.g., Option 1 in **Figure 1**). In Study 1, 9% of preschoolers, 11% of elementary schoolers, and 29% of adults followed the majority systematically in choices in this pattern type (i.e., 6 out of 8). In Study 2, 6% of preschoolers, 12% of elementary schoolers, and 20% of adults followed the majority systematically in choices. Accordingly, the proportion of individuals who may have applied an Equal Weight Rule is low, especially in child groups.
